# Supplementary material for: Gerstmann–Sträussler–Scheinker Disease with F198S Mutation Induces Independent Tau and Prion Protein Pathologies in Bank Voles
Source: Biomolecules. 2022 Oct 21;12(10):1537. doi: 10.3390/biom12101537 (PMC9599806; doi:10.3390/biom12101537)
Supplement: Supplementary file 1 [file biomolecules-12-01537-s001.zip › biomolecules-1884747-supplementary.pdf]

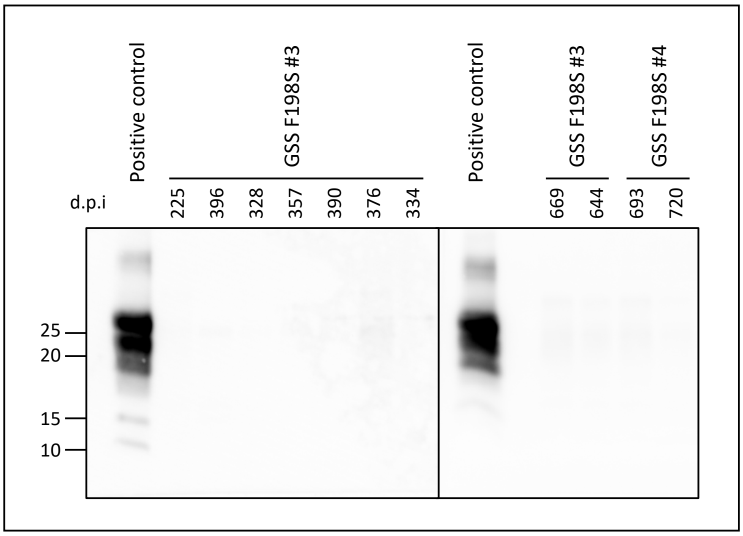

Figure S1. Western blot analysis showing the absence of PrP^{Sc} in brains of BvM inoculated with GSS-F198S cases. Brain homogenates from voles inoculated with GSS-F198S case #3 or #4 (as indicated on the top of the blots) were treated with PK (at a final concentration of 50 μg/ml) and analyzed with a mix of SAF84 and 9A2 mAbs, in order to detect both C-terminal and N-terminal PrP^{res} fragments. The blots show the analysis of representative animals sacrificed for intercurrent disease or found dead at different d.p.i. (days post inoculation) as indicated in the top of the blots. MW markers, indicated on the left, represent 25, 20, 15 and 10 kDa. Tissue equivalents (TE) loaded per lane were 0.5 mg for all samples, including the positive control (vole-adapted sCJD-MV1) loaded in the first lane of each blot.

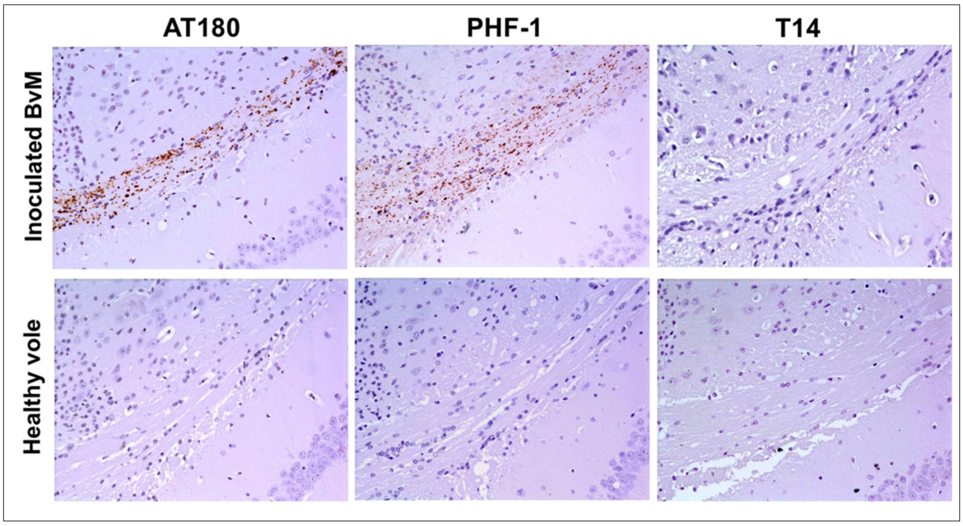

Figure S2. Filamentous Tau pathology in BvM inoculated with GSS-F198S. Using different anti-Tau antibodies, AT180 and PHF-1, respectively against pT231 and pS396/pS404 epitopes, the same pTau pattern deposition was observed. pTau-positive material detected in voles was negative when analyzed with human Tau-specific T14 antibody.

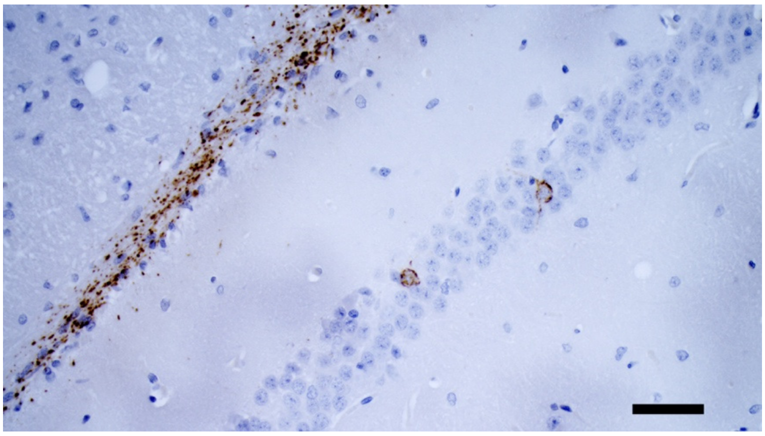

Figure S3. Tau pathology in bank voles inoculated with fAD-PS1. Hyperphosphorylated Tau aggregates in the hippocampus of a BvM culled at 810 d.p.i., inoculated with fAD-PS1. In the picture are visible Bv-pTau inclusions accumulated along the alveus as neuropil threads and coiled bodies. Two immunoreactive neuronal bodies are detected in CA1. Immunostaining was performed with AT8 antibody (scale bar 20 μm).

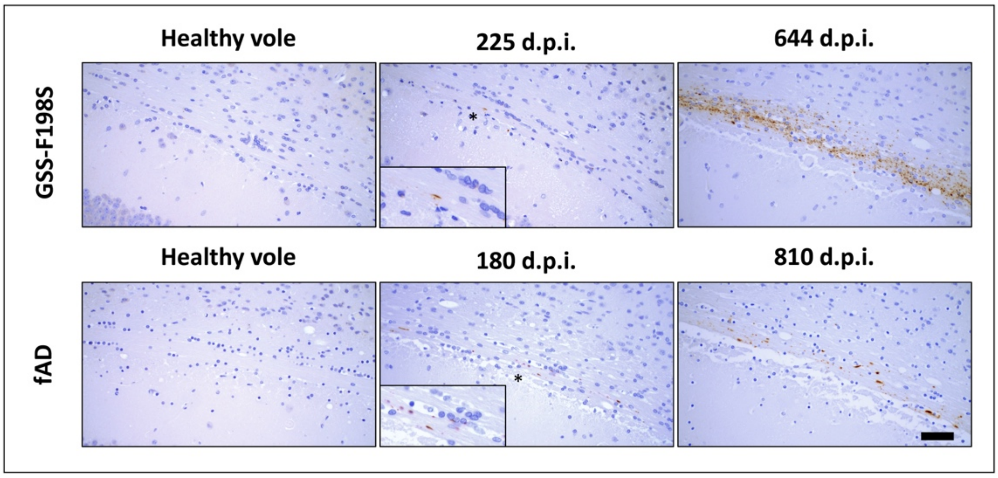

Figure S4. Variable density of bv-pTau deposition over the time. The vole inoculated with GSS-F198S #3 and culled at 225 d.p.i. and voles inoculated with fAD and culled at 180 d.p.i. displayed a low amount of Bv-pTau deposits in comparison with voles culled or succumbed successively showed high deposition density. Immunostaining was performed with AT8 antibody (scale bar 20 μm).
